# Polymorphisms in the hypoxia-inducible factor 1 alpha gene in Mexican patients with preeclampsia: A case-control study

**DOI:** 10.1186/1756-0500-4-68

**Published:** 2011-03-17

**Authors:** Sonia Nava-Salazar, Elly N Sánchez-Rodríguez, C Adriana Mendoza-Rodríguez, Carlos Moran, Juan F Romero-Arauz, Marco A Cerbón

**Affiliations:** 1 Departamento de Biología, Facultad de Química, Universidad Nacional Autónoma de México, México D.F. 04510, México; 2 Hospital de Ginecología y Obstetricia 4, Luis Castelazo Ayala, Instituto Mexicano del Seguro Social, México D.F. 01090, México

## Abstract

**Background:**

Although the etiology of preeclampsia is still unclear, recent work suggests that changes in circulating angiogenic factors play a key role in its pathogenesis. In the trophoblast of women with preeclampsia, hypoxia-inducible factor 1 alpha (HIF-1α) is over-expressed, and induces the expression of non-angiogenic factors and inhibitors of trophoblast differentiation. This observation prompted the study of HIF-1α and its relation to preeclampsia. It has been described that the C1772T (P582S) and G1790A (A588T) polymorphisms of the *HIF1A *gene have significantly greater transcriptional activity, correlated with an increased expression of their proteins, than the wild-type sequence. In this work, we studied whether either or both *HIF1A *variants contribute to preeclampsia susceptibility.

**Results:**

Genomic DNA was isolated from 150 preeclamptic and 105 healthy pregnant women. Exon 12 of the *HIF1A *gene was amplified by PCR, and the genotypes of *HIF1A *were determined by DNA sequencing.

In preeclamptic women and controls, the frequencies of the T allele for C1772T were 4.3 vs. 4.8%, and the frequencies of the A allele for G1790A were 0.0 vs. 0.5%, respectively. No significant differences were found between groups.

**Conclusion:**

The frequency of the C1772T and G1790A polymorphisms of the *HIF1A *gene is very low, and neither polymorphism is associated with the development of preeclampsia in the Mexican population.

## Background

Preeclampsia (PE), a systemic syndrome occurring in pregnant women, is characterized by the new onset of hypertension and proteinuria after 20 weeks of gestation and prior to 48 h postpartum [1-3]. It is the most important cause of morbidity and mortality in the mother-fetus binomial, affecting 5 to 8% of pregnant women worldwide. The actual percentage depends on the population studied and the definition of preeclampsia employed [4-6].

One of the main characteristics of PE is an inadequate trophoblast invasion leading to an incomplete remodeling of the spiral artery, a reduction in utero-placental perfusion, and a state of placental hypoxia. It is considered that this condition can trigger widespread maternal endothelial dysfunction, and therefore the systemic manifestation of PE [7,8].

HIF-1α is the major transducer of hypoxia signaling in several tissues, including human placenta [9,10]. Several studies suggest that hypoxia-inducible factor 1 alpha (HIF-1α), and two of the numerous genes that it regulates [11], soluble fms-like tyrosine kinase 1 (sFlT-1) and soluble endoglin (sEng) [12,13], are over-expressed in preeclamptic women and play a key role in the development of PE [14-17].

It has been reported that a base change of C to T at 1772, or G to A at 1790 in exon 12 of the *HIF1A *gene can increase the transcriptional activity of this gene compared to the wild type isoform [18]. Several studies have reported associations between these polymorphisms and different diseases in which alterations of HIF-α and its target genes are implicated, such as diabetes, coronary disease and cancer (renal cell carcinoma; head, neck, colorectal, breast, and lung cancer) [19-22].

The aim of this study was to determine if there is an association between the presence of C1772T and/or G1790A polymorphisms and the development of PE in the Mexican population.

## Methods

### Population

We conducted a prospective case-control study with patients admitted to the Gynecology Hospital 4, Luis Castelazo Ayala of Mexican Social Security Institute in Mexico City. Patients were recruited from September 2008 to July 2010, including 105 healthy pregnant women and 150 preeclamptic women. Among the latter group, 29 patients had mild PE (MPE) and 121 severe PE (SPE). 36 patients presented HELLP syndrome and 11 intrauterine growth restriction (IUGR).

The diagnosis of PE was made according to international and Mexican Ministry of Health guidelines [1,3]. Criteria for MPE were as follows: blood pressure ≥ 140/90 mm Hg persisting for at least 6 h, and proteinuria ≥300 mg/day after the 20th week of gestation. Criteria for SPE were: blood pressure ≥ 160/110 mmHg persisting for at least 6 h, and proteinuria ≥2 g/day after the 20th week of gestation. Additionally, at least one of the following symptoms was present for SPE: headache, visual disturbance, epigastric pain or pulmonary edema. Criteria for HELLP syndrome were: thrombocytopenia (≤100 000/μl), increase in lactic dehydrogenase (LDH) (>600 U/l), aspartate aminotransferase and alanine aminotransferase levels (AST and ALT ≥70 U/l). IUGR was determined by a birth weight below the 10th percentile for gestational age. Women with term pregnancy and without complications were recruited as controls. Excluded from the study were women with chronic hypertension, diabetes mellitus, gestational diabetes, renal or autoimmune disease.

The information on maternal demographic characteristics and family history was gathered from all subjects by personal interview. The study was approved by the Ethics Committee of the Hospital and by the Mexican Ministry of Health. Informed consent was obtained from all patients and controls.

### Genotyping

Genomic DNA was isolated from the peripheral blood leukocytes by using standard protocols. Polymerase chain reaction (PCR) was performed to amplify the 178-bp fragment of the exon 12 of the *HIF1A *human gene, as previously reported [18], using the 5'-CAT GTA TTT GCT GTT TTA AAG-3' forward primer and 5'-GAG TCT GCT GGA ATA CTG TAA CTG-3' reverse primer. The mixture for PCR was in 30 μL, containing 200 ng template DNA, 0.2 mM of each dNTP, 0.5 μM of each forward and reverse primer, 1.5 mM MgCl_2_, 0.5 U of Taq polymerase and 3 μL of 10X PCR buffer. The conditions for the PCR reaction were: denaturation at 95°C for 5 min, followed by 35 cycles of denaturation at 95°C for 30 sec, annealing at 61°C for 30 sec, extension at 70°C for 1 min, and a final extension at 72°C for 10 min. PCR products were purified and sequenced using Big Dye Terminator kit (version 3.1) on an ABI Prism^® ^3100 Automated DNA sequencer according to the manufacturer's protocol (Applied Biosystems, Foster City, CA).

### Statistical analysis

We compared clinical characteristics between preeclamptic and control groups. Data are presented as the mean ± SEM. Statistical significance between groups was established with a one-way analysis of variance (ANOVA), followed by the Turkey's multiple comparison test or student-*t *test, as appropriate.

Differences in genotypic frequencies between the two groups were tested for significance by using two by two contingency tables and Fisher's exact statistical test, and the odds ratios were calculated using 95% confidence intervals (CI). Data were analyzed using Graph Pad Prism Software V.5.1.

Test for the Hardy-Weinberg equilibrium, which revealed normal distribution of the population, was performed by the χ^2 ^test in http://www.oege.org.

The frequencies of genetic polymorphisms and the prevalence of PE in Mexican population (reported as 7%) were used for statistical power calculation in Quanto V.1.2.

P-values <0.05 were considered as statistically significant. All statistical tests were two-tailed.

## Results

We conducted a case-control study to determine whether the presence of either of two single nucleotide polymorphisms (SNP) of the *HIF1A *gene is associated with the development of PE in Mexican population.

Table 1 shows the demographic and clinical characteristics of cases and controls. The group of women with PE showed a lower infant birth-weight, shorter gestation, and higher body mass index (BMI) compared to the control group. There were also a higher percentage of women with PE who smoked before pregnancy. All of these differences were statically significant. There were no differences in age and the frequency of primigravidas between the two groups. The seventeen PE patients (18%) who were multigravidas had a past history of PE.

**Table 1:** Demographic and clinical characteristics of PE patients and the control group

Parameters	Control	PE		P-value
N	105	150		
Maternal Age (years)	27.7 ± 0.6	28.4 ± 0.6		NS
Smoking before pregnancy (%)	16.2	27.7		0.034
Primigravid (%)	37.9	36.9		NS
BMI (Kg/m^2^)	28.9 ± 0.5	31.3 ± 0.8		0.011
Newborn weight (g)	2991.0 ± 63.1	2177.0 ± 74.6		< 0.001
GAD (weeks)	38.2 ± 0.2	34.8 ± 0.3		< 0.001

		**MPE**	**SPE**	

SBP (mmHg)	114.1 ± 0.7	137.7 ± 1.9	155.0 ± 1.4	< 0.001 ^♦^
DBP (mmHg)	73.3 ± 0.6	89.7 ± 1.3	100.9 ± 1.0	< 0.001 ^♦^
Proteinuria (mg/24 h)	ND	443.6 ± 63.5	2482.0 ± 377.4	< 0.001 ^♦♦^

Values are the mean ± SEM. PE, preeclampsia; BMI, body mass index; GAD, gestational age at delivery; MPE, mild preeclampsia; SPE, severe preeclampsia; SBP, systolic blood pressure; DBP, diastolic blood pressure; ND: not determined but negative using dipstick test. P values were calculated between the control and preeclampsia group. NS, non significant. ^♦ ^Control vs. MPE and SPE; MPE vs. SPE. ^♦♦ ^MPE vs. SPE

The genotypic frequencies of C1772T and G1790A polymorphisms of the *HIF1A *gene are presented in Table 2. There were no significant differences in the distribution of the two polymorphisms between groups. None of the individuals presented the two polymorphisms, and those with either of the polymorphisms were considered heterozygotes. In the group of cases with C1772T polymorphism, 3 patients presented MPE and 10 SPE. Two of the latter also had IUGR and one had HELLP syndrome. The G1790A polymorphism was only observed in one women of the control group, also considered heterozygote. The genotype frequencies were in agreement with the Hardy-Weinberg equilibrium.

**Table 2:** Genotype and allele frequencies of the C1772T and the G1790A polymorphisms of the *HIF1A *gene in PE patients and the control group

Nucleotide	Amino acids	Genotypes	PE (%)	Control (%)	P-value	OR (95% CI)
**C1772T**	**Pro582**	CC	137 (91.3)	95 (90.5)		
	**Ser582**	CT	13 (8.7)	10 (9.5)		
		TT	0 (0.0)	0 (0.0)		
		CT + TT	13 (8.7)	10 (9.5)	0.203 *	0.485 (0.178-1.067)
		**Allele**				
		C	287 (95.7)	200 (95.2)	0.831	0.906 (0.389-2.107)
		T	13 (4.3)	10 (4.8)		

		**Genotypes**				

**G1790A**	**Ala588**	GG	150 (100.0)	104 (99.3)		
	**Thr588**	GA	0 (0.0)	1 (0.7)		
		AA	0 (0.0)	0 (0.0)		
		GA + AA	0 (0.0)	1 (0.7)	0.412 **	0.232 (0.009-5.737)
		**Allele**				
		G	300 (100.0)	209 (99.5)	0.411	0.232 (0.009-5.737)
		A	0 (0.0)	1 (0.5)		

PE, preeclampsia; OR, odds ratio; CI, confidence intervals. * Calculations were performed CC vs. CT + TT. ** Calculations were performed GG vs. GA + AA

## Discussion

There is strong evidence that an imbalance between angiogenic factors, such as vascular endothelial growth factor (VEGF), placental growth factor, and factors inhibiting angiogenesis, such as sFlt1 and sEng, are closely related to the pathogenesis of PE [16,17]. Since HIF-α is the main regulator of the angiogenic/anti-angiogenic factors that are over-expressed in women with PE, there has been growing interest in the biology of the HIF-α pathway and its role in human placenta development [14].

I*n vitro *studies have demonstrated that under both normoxic and hypoxic conditions, the C1772T (P582S) and G1790A (A588T) polymorphisms of the *HIF1A *gene has a higher transcriptional activity, correlated with an over-expression of the corresponding protein, compared to the transcriptional activity and protein expression of the wild-type sequence [18,23,24]. Furthermore, in various groups among different populations, an association has been found between the presence of these polymorphisms and the development of diseases in which an altered HIF-1α is implicated, such as cancer, diabetes and coronary disease [18-22].

In this study we determined the frequency of the C1772T and G1790A polymorphisms of the *HIF1A *gene and its possible correlation with PE risk in Mexican population.

Analyzing the clinical and demographic characteristic of our population (Table 1), we did not find a significant difference in age and parity between PE patients and women with normal pregnancies. However, it has been reported that women ≥ 40 years old have twice the risk of PE, whether they were primiparous or multiparous, and that as of the age of 34 the risk increases by 30% for each additional year. Nulliparous women have triple the risk of developing PE, especially in young women (≤20 years old) [25,26]. It is likely that the lack of significant differences between our study groups in relation to risk for PE is due to the age range of women included in the study.

Cigarette smoke exposure is associated with many adverse effects in pregnancy, such as preterm labor, preterm premature rupture of membranes, placental abruption and IUGR. Paradoxically, smoking has been associated with a decreased risk of PE. Several studies argue that it has a protective effect if women continue to smoke during pregnancy, and that contrarily, the protective effect is lost if the mother stops smoking [27,28]. In our study, the percentage of smokers in the PE group was higher compared with the control group, a difference that was statistically significant (P = 0.034, OR = 1.984). According to the questionnaires applied to patients, in all cases the women stopped smoking before pregnancy. Thus, the lack of protective effect under these circumstances is in agreement with the reported data.

With regard to body mass index, several studies suggest that obese women have twice the risk of developing PE, compared to women with normal BMI (20 -25). Women with BMI >35 before pregnancy had over four times the risk of PE [29]. The PE group had an average BMI of 31, classified as obese type I, which was statistically different with regard to the BMI of the control group.

There were also significant differences between the two groups in terms of gestational age at delivery (GAD) and newborn weight. 59% of women of the preeclamptic group delivered before 34 weeks of gestation, a proportion that was higher among women with SPE (63%) than MPE (41%). The tendency to an early delivery is due to the fact that PE is characterized by progressive deterioration in both maternal and fetal conditions. For this reason, studies and clinical practice indicate that after 34 weeks, the risk of continuing the pregnancy is greater than the benefit, resulting in the decision to terminate the pregnancy and the consequent low weight of newborns. Additionally, PE can be complicated with IUGR (10-25%). With this condition, the physiological condition of the fetus determines whether the pregnancy is continued or terminated [2]. PE is a heterogeneous disease, and therefore the differences between these factors depend on the individual condition of each patient.

The genetic analysis of HIF polymorphisms between the patients with PE and women with normal pregnancies indicates that there are no significant differences in terms of the distribution of C1772T and G1790A genotypes and alleles (Table 2). For the 1772T allele, we found frequencies similar to those reported for other populations, such as Japan, Finland, Korea and China [18,21,22,30-32]. The frequency of homozygous for the T allele is very low (0.010-0.031). In some populations, like ours, the homozygous has not been found, which may be due to its low frequency per se in the Mexican population and/or the size of the sample analyzed.

In our population the frequency of the 1790A allele was 0.005. This value is low compared to other populations (0.015 - 0.047). Indeed, the homozygote for the A allele is very rare worldwide, and has only been reported in Japanese and Korean populations [19,21].

There is an ethnicity-related variation in the distribution of C1772T and G1790A polymorphisms, as reflected by studies of its association with particular diseases in different populations [21,33].

In a study of both these polymorphisms and their association with the development of PE in a Finnish population [30], the frequency of the C1772T polymorphism was similar to that found in our study. Contrarily, our results show that the frequency of the G1790A variant was lower than in the Finnish population. Nevertheless, in neither the Finnish study nor in our own, any association was found between the presence of these polymorphisms and the development of PE.

To the best of our knowledge, the Finnish study and the current contribution are the only ones to evaluate the frequency of the P582S and A588T polymorphisms of *HIF1A *gene in patients with PE. Therefore, the present study represents the first contribution from a Latin American population. One limitation of this study is the low statistical power; as the sample size provides a power less than the 50% under a dominant model. On the one hand, the sample size should be increased, however, from our point of view, due to the insignificant statistical difference observed between the study groups, mainly for the variant P582S, these results would not change if a larger number of patients were included in each study group.

The inability to develop satisfactory prevention and clinical prediction strategies for PE has led to various focuses of investigation. Since previous reports have suggested that changes in circulating angiogenic factors are strongly related to the pathogenesis of PE, one of the approaches has been to attempt to establish a predisposition to this disease in relation to genes that are involved in the angiogenesis pathway. The result of association studies between PE and different angiogenic genes, such as *VEGF*, *FLT *and *ENG*, are inconsistent [34-37]. Hence, further studies are necessary to determine whether these polymorphisms and their proteins play a key role in the development of PE.

## Conclusion

In the Mexican population, C1772T and G1790A polymorphisms of the *HIF1A *gene are not associated with the development of PE.

## Competing interests

The authors declare that they have no competing interests.

## Authors' contributions

SNS, the expert in the field and major contributor to the manuscript, performed the experiments, analyzed data and drafted the manuscript. ENSR helped in collecting and processing biological samples. CAMR reviewed and participated in the design of the experiments. CM and JFRA participated in the study by diagnosis, selection of the studied population and medical care of the patients. MAC participated in the overall study design, project oversight and critical review of the manuscript. All authors read and approved the final manuscript.
